# Pneumococcal Serotypes and Serogroups Causing Invasive Disease in Pakistan, 2005–2013

**DOI:** 10.1371/journal.pone.0098796

**Published:** 2014-06-03

**Authors:** Sadia Shakoor, Furqan Kabir, Asif R. Khowaja, Shahida M. Qureshi, Fyezah Jehan, Farah Qamar, Cynthia G. Whitney, Anita K. M. Zaidi

**Affiliations:** 1 Department of Pediatrics and Child Health, the Aga Khan University Hospital, Karachi, Pakistan; 2 Department of Pathology and Microbiology, the Aga Khan University Hospital, Karachi, Pakistan; 3 National Center for Immunization and Respiratory Diseases, CDC, Atlanta, Georgia, United States of America; University of Cambridge, United Kingdom

## Abstract

While pneumococcal conjugate vaccines have been implemented in most countries worldwide, use in Asia has lagged in part because of a lack of data on the amount of disease that is vaccine preventable in the region. We describe pneumococcal serotypes elicited from 111 episodes of invasive pneumococcal disease (IPD) from 2005 to 2013 among children and adults in Pakistan. Seventy-three percent (n = 81) of 111 IPD episodes were cases of meningitis (n = 76 in children 0–15 years and n = 5 among adults). Serotypes were determined by target amplification of DNA extracted from pneumococcal isolates (n = 52) or CSF specimens (n = 59). Serogroup 18 was the most common serogroup causing meningitis in children <5 years, accounting for 21% of cases (n = 13). The 10-valent pneumococcal conjugate vaccine (PCV 10) or PCV10- related serotypes were found in 61% (n = 47) of childhood (age 0–15 years) meningitis episodes. PCV-13 increased this coverage to 63% (one additional serotype 19A; n = 48). Our data indicate that use of PCVs would prevent a large proportion of serious pneumococcal disease.

## Introduction

*Streptococcus pneumoniae* is a major cause of childhood mortality worldwide. The World Health Organization estimates that approximately 0.5 million deaths among HIV-negative children <5 years were attributed to pneumococcal disease in 2008 [1]. Up to 80% of these deaths occurred in children residing in the developing world [2]. With the advent of pneumococcal conjugate vaccines (PCV), invasive pneumococcal disease (IPD) and pneumococcal pneumonia in children are now preventable illnesses. However, reduction in rates of IPD is dependent on the proportion of disease caused by vaccine serotypes. Although current PCV10 and PCV13 formulations include the most common serotypes causing IPD among children worldwide [3], complex epidemiology and variation of strains among geographical regions suggest that gaps may exist in vaccine coverage for prevalent pneumococcal serotypes circulating in some populations [4]. Limited recent data from South East Asia suggests that the proportion of IPD covered by PCV might be lower in this region than elsewhere [5]. IPD surveillance in Bangladesh has shown that from 2004 to 2007 only 38% of meningitis and 42% of nonmeningitis infections were covered by PCV10 [6].

Mastro et al reported pneumococcal serotypes causing pneumonia in 87 IPD cases from 1986–1989 in Rawalpindi, Pakistan [7]. Serotype 19F was found to be the most common serotype causing pneumonia among children (n = 28, 32.2%), followed by serotype 31 (n = 13, 14.9%), serotype 16 (n = 12, 13.8%), serotype 19A (n = 11, 12.6%), serotype 9V (n = 9, 10.4%), serotype 6A (n = 4, 4.6%), serotype 15C (n = 4, 4.6%), serotype 5 (n = 2, 2.3%), and each of serotypes 1, 6B, 9A, and 18A (n = 4, 4.6%). Only 47.1% of these serotypes are covered by PCV 10. Since the publication of this data in 1991, no further studies have been undertaken. To provide more recent data on the potential for PCV to reduce disease in the region, we report pneumococcal serotypes elicited by PCR in 111 IPD episodes in Pakistan from 2005–2013.

## Methods

### Data sources

Isolates were obtained from several sources. From 2005 to 2008, pneumococci isolated from routine microbiological culture of sterile body fluids from both adults and children were cryopreserved at −70°C at the Aga Khan University Infectious Disease Research Laboratory and serotyped. In 2009, Johns Hopkins Bloomberg School of Public Health funded a meningitis surveillance project through the GAVI Alliance's Hib Initiative to study the impact of introduction of *Haemophilus influenzae* type b (Hib) vaccine in Pakistan. In this study, cerebrospinal fluid (CSF) specimens were obtained from children less than 5 years of age at sentinel surveillance sites in lower Sindh. Surveillance sites included secondary and tertiary care hospitals in the districts of Karachi, Matiari, and Hyderabad, with an overall population of 21.5 million [8], [9], with 60–80% of the population residing in urban areas. CSF samples collected in the study were subjected to polymerase chain reaction (PCR)-based detection of common agents of purulent meningitis. Culture was not performed because of our previous experience of low yield, as the vast majority of CSF specimens are obtained after initiation of antimicrobial therapy. This study concluded in 2013. Of 288 samples obtained till February 2013, 59 samples positive for pneumococci were serotyped. A further 5 pneumococcal bloodstream isolates were obtained from two ongoing neonatal sepsis surveillance studies in Karachi (four cases from 2010, and one case from 2013) and 2 bloodstream isolates were obtained from an ongoing pneumonia surveillance study in the Hyderabad district of lower Sindh (one isolate each from 2012 and 2013).

All primary studies were approved by the Aga Khan University Ethical Review Committee. CSF and blood samples were collected after obtaining written informed consent from parents or guardians of children.

(The collection of CSF samples and use for further typing was approved in the primary Hib study by the Aga Khan University Ethics Review Committee ERC Approval No. 869-Ped/ERC-07. This is the study contributing the largest number of samples to this dataset).

### Culture of pneumococci, colony identification and DNA extraction

Standard protocols as described elsewhere [10] were used for culture of sterile body fluids (blood, CSF, ascitic, or pleural fluid). Pneumococcal isolates were identified by optochin sensitivity and bile solubility tests. Isolates archived in 15% glycerol with buffered phosphate and 4 mm glass beads were revived on blood agar media and re-identified before PCR serotyping, following the same procedures.

DNA was isolated from pneumococcal isolates by boiling. Briefly, a loop of pneumococcal colonies were suspended in 1 ml of nuclease-free water and heated to 100°C for 10 minutes. Lysates were stored at −20°C till further use.

### Processing of CSF samples – DNA extraction, detection of *Streptococcus pneumoniae*

From CSF specimens, DNA was extracted using the QIAmp DNA mini kit (QIAGEN Inc. Valencia, Calif.) according to the manufacturer's instructions (Spin protocol). Samples were pretreated by suspending 20 µl of CSF in 100 µl TE buffer, 0.04 gm/ml lysozyme, and 75 U/ml mutanolysin and incubating the mixture at 37°C for 1 hour. Thereafter, the protocol described for the QIAmp DNA mini kit by the manufacturer was followed.

Following DNA extraction from CSF samples, *S. pneumoniae* was detected by a multiplex real time PCR for *S. pneumoniae*, *H. influenzae*, and *Neisseria meningitidis* by targeting genes *lyt*A, *bex*A,and *ctr*A, respectively [11]. Briefly, a 25 µl reaction volume was used with 10 µM each of forward and reverse primers and 5 µM of probe for each respective gene. Triplex real-time PCR was performed in Corbett Rotor-Gene 6000 (Corbette Life Science, USA) with the following cycling conditions: 50°C for 2 minutes, 95°C for 10 minutes, followed by 50 cycles at 95°C for 15 s and 60°C for 1 minute. A Ct value of <35 was considered positive. All runs incorporated a negative water control and *S.pneumoniae* ATCC 49619 template DNA as positive control. DNA extracted from CSF specimens was also archived at −20°C.

### PCR serotyping

Sequential multiplex PCR serotyping was performed on DNA extracted either from bacterial isolates or from CSF samples as described by Pai et al [12]. Thirty-three primer pairs were grouped into 12 multiplex reactions for serogroups/serotypes 1, 3, 4, 5, 6A/B/C, 7B/C/40, 10A, 11A/D, 12F/A/44/46, 15A/F, 15B/C, 17F, 18A/B/C/F, 19F, 20, 22A/F, 23F, 33F/A/37, 35F/47F (Pai et al) [12], 19A (Pimenta et al) [13], 2, 7A/F, 8, 9V/A, 10F/C/33C, 13, 23A, 23B, 24A/B/F, 35A/C/42, 39 (Carvalho et al) [14], and 14, 9N/L (Dias et al) [15]. The primer for the conserved pneumococcal polysaccharide capsule gene (*cps*A) was added as control with each multiplex reaction. Non-typeable samples were also subjected to PCR serotyping for the following additional serotypes/serogroups in monoplex reactions: 6C/6D, 16F, 21 (Carvalho et al) [14], and 31, 34, 35B, and 38/25F/25A (Pai et al) [12].

Multiplex reactions contained primers for 2–4 serotypes/serogroups and positive internal control *cps*A. PCR was carried out in reaction mixture volumes of 25 µl (22 µl for monoplexes), with 25 µM primers, and 2X multiplex master mix (Qiagen). Amplification was performed in Eppendorf Master Cycler (Eppendorf, Hamburg, Germany) with the following cycling conditions: 95°C for 15 minutes, then 35 cycles of 95°C for 30 s, 54°C for 90 s, and 72°C for 60s. PCR final extension was performed at 72°C for 10 minutes. PCR products were electrophoresed on 2% agarose gel stained with SYBR Green (Sigma, USA) and visualized under UV light (GEL-DOC molecular imager, Biorad Inc. USA).

Serotypes 1, 3, 4, 5, 14, 19A, 19F, and 23F (contained within PCV 10 and PCV 13) were defined as vaccine serotypes (V). Serotypes contained within serogroups 18A/B/C/F, 7A/F, 9V/A, and 6A/B/C were classified as vaccine-related (VR) as these could not be further resolved to individual serotypes by PCR serotyping. Serotypes contained within the same serogroup as vaccine serotypes were also classified as vaccine related. All other serogroups and serotypes (including non-typeable) were classified as non-vaccine serotypes (NV).

## Results

Serotypes were elicited for 111 episodes of IPD from 2005 to 2013. Of these, 76 (68.4%) episodes were cases of meningitis (72 cases in children 0–59 months of age), while 17 were cases of non-meningitis IPD (NM-IPD) in children aged 0–15 years. Overall, 84 (75.6%) cases were from children under 5 years. Eighteen (n = 18; 16.2%) strains of pneumococci isolated from IPD in adults and cryopreserved from 2005 to 2008 were also serotyped. Table 1 describes IPD cases, sources and sample types, and serotypes determined in all 111 episodes.

**Table 1:** IPD serotypes from Pakistan by age group and specimen source, 2005–2013.

	Age group and syndrome	Source	Number of cases		Serotypes (n)	Proportion of serotypes included in PCV-10* (%)	Proportion of serotypes included in PCV-13 (%)
	**0–59 months**						
	**Meningitis**	CSF	72			47/72 (65.3%)	48/72 (66.7%)
				13 (2005–6)	19F (4), 18A/B/C/F (2), 11A/D (2), 9V/A (1), 15B/C (1), 22A/F (1), 1 (1), NT†(1)	8/13 (61.5%)	8/13 (61.5%)
				59 (2009–13)	18A/B/C/F (11), 14 (7), 19F (2), 23B (6), 12F/A/44/46 (6), 5 (4), 9V/A (2), 1(1), 15B/C (1), 10A (2), 6A/B/C (2), 4 (2), 23F(2), 19A (1), 17 (1), 8 (1), 9N/L (1), 24A/B/F (1), 33F/A/37 (1), 35B (1), NT† (4)	39/59 (66.1%)	40/59 (67.8%)
	**Sepsis**	Blood	12		5 (3), 1 (2), 23A (2), 23F (1), 3 (1), 14 (1), 10F/C/33C (1), 35B (1)	9/12 (75%)	10/12 (83.3%)
**Subtotal**			**84**			**56/84 (66.7%)**	**58/84 (69%)**
	**5–15 years**						
	**Meningitis**	CSF	4		7A/F (1), 11A/D (1), 24A//B/F (1), NT (1)	0/4 (0%)	0/4 (0%)
	**Sepsis**	Blood	3		18A/B/C/F (1), 1 (1), 19F (1)	3/3 (100%)	3/3 (100%)
	**Empyema**	Pleural fluid	2		19F (2)	2/2 (100%)	2/2 (100%)
**Subtotal**			9			**5/9 (55.6%)**	**5/9 (55.6%)**
	**Adults (18–70 years)**						
	**Meningitis**	CSF	5		5 (1), 13 (1), 19F (1), 22A/F (1), NT (1)	2/5 (40%)	2/5 (40%)
	**Sepsis**	Blood	8		1 (2), 23F (2), 13 (1), 15B/C (1), 38/25F/25A (1)	4/8 (50%)	4/8 (50%
	**Other** **	See below	5		19F (3), 1 (1), 14 (1)	5/5 (100%)	5/5 (100%)
**Subtotal**			**18**			**11/18 (61.1%)**	**11/18 (61.1%)**
**TOTAL**			**111**			**72/111 (64.9%)**	**74/111 (66.7%)**

*Includes Vaccine -related (VR) serotypes.

† NT =  Non-typeable.

**Ascitic fluid (n = 3), synovial fluid (n = 1), unknown sterile body fluid (n = 1).

### IPD serotypes in children 0–59 months

**Figure 1:**
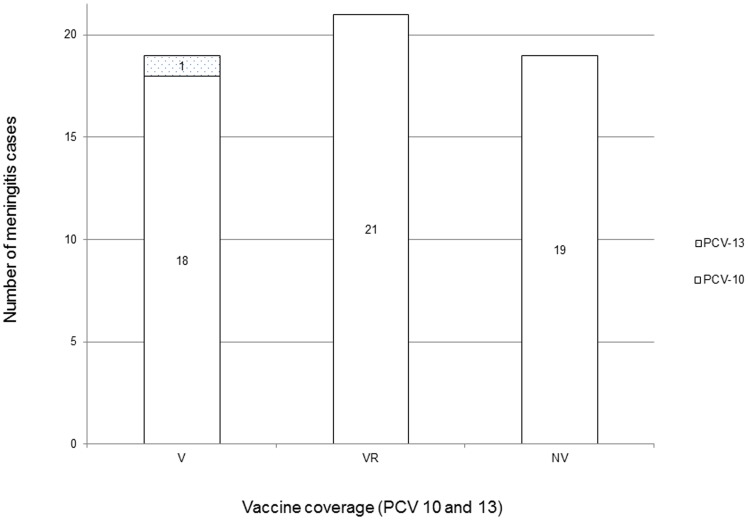
Vaccine coverage of pneumococcal serotypes in meningitis, Karachi and Hyderabad, Pakistan; 2009–2013 in children 0–59 months. (V =  Vaccine serotypes, VR =  Vaccine-related serotypes, and NV =  non-vaccine serotypes).

Serotyping by PCR was performed on 72 individual meningitis cases. Of these, 13 cases were taken from 2005–2006 (with archived pneumococcal isolates), and 59 cases were obtained from the Hib meningitis study [9]. The most common serogroup causing meningitis was serogroup 18 (n = 13), followed by serogroup 14 (n = 7), serotype 19F (n = 6), and serotype 23B (n = 6). The non-vaccine serotype 12F/A (detected as 12F/A/44/46) was found in 6 cases. Five cases remained non-typeable by PCR. These 5 were positive for *lyt*A (detected in the CSF triplex) as well as for *cps*A genes. Serotype data has been temporally disaggregated in Table 1 and Figure S1 in File S1. Figure 1 shows the proportion of strains covered by PCVs among children 0–59 months with meningitis seen from 2009 to 2013.

Bacterial isolates were serotyped from five cases of neonatal sepsis and seven cases of sepsis in children 1–59 months old (Table 1). Four of five cases of neonatal sepsis were caused by PCV-10 vaccine serotypes while one case was due to serotype 3, which is contained in PCV-13. Temporal distribution of sepsis serotypes is shown in Figure S2 in File S1.

Overall, 66.7% of IPD serotypes from children less than 5 years are those covered by PCV 10 (vaccine or vaccine-related serotypes), including 65.3% (47 of 72) of meningitis and 75% (9 of 12) of non-meningitis cases. An additional 2 cases with one serotype 19A in meningitis and one case with serotype 3 in neonatal sepsis were potentially preventable with PCV-13, increasing overall coverage from 66.7% to 69%.

### IPD serotypes in children 5–15 years and adults

Nine pneumococcal isolates from children 5–15 years of age and 18 isolates from adults (2005–2008) were available for serotyping. Among the 27 strains, 16 (59.3%) were serotypes included in PCV10 (Table 1).

## Discussion

This is the first time pneumococcal serotypes responsible for childhood meningitis are being reported from Pakistan, adding to earlier data from Mastro et al, who reported serotypes from pneumonia cases among children [7]. Although IPD is not a rare illness, the complex epidemiology and surveillance difficulties have made estimation of IPD incidence challenging. Zaidi et al reported the incidence of pneumococcal meningitis as 81 per 100 000 infants less than 1 year of age and 20 cases per 100 000 children less than 5 years of age in 2005 [16]. Considering this high incidence and case fatality rates of up to 50% for pneumococcal meningitis [17], understanding the pneumococcal serotypes responsible for meningitis and the proportion likely to be preventable by vaccine are valuable data for policymakers in Pakistan and in similar countries in the region as PCV10 and PCV13 are being introduced or being considered for introduction.

PCV 7 (Wyeth, now Pfizer) was introduced in Pakistan for the private market in May 2006 (Pfizer, data on file). Vaccination rates, however, have been low and inconsistent due to unaffordable vaccine prices and inaccessibility of private pharmacy services. We therefore consider the community data presented here as reflecting the serotype distribution of a vaccine-naïve population. PCV10 was introduced in Pakistan as part of its Expanded Program on Immunizations in March 2013 with support from the GAVI Accelerated Vaccine Introductions initiative. This report describes pneumococcal serotype distribution in IPD in Pakistan from before March 2013. Given data from Bangladesh showing vaccine coverage closer to 40%, an overall serotype coverage of 65.6% (PCV-10) for childhood IPD (61 of 93 serotypes) is reassuring in the wake of vaccine introduction and roll-out. However, data from continued IPD surveillance after the PCV10 roll-out will be a better indicator of vaccine efficacy in the Pakistani pediatric population.

Serotype proportions we report differ from those reported by Mastro et al [7] for pneumococcal pneumonia. Serotype 19F was the most common serotype reported by Mastro, whereas only 9.7% of meningitis cases in children are caused by 19F. Furthermore, serotypes 31 and 16 were also commonly seen in pneumonia, but were not found in meningitis cases. However, this comparison is confounded by temporal as well as regional differences as well as possible circulation of outbreak strains. Data should therefore be interpreted keeping in view these important differences.

We found most pneumococcal meningitis to be due to serogroup 18 (18A/B/C/F). Individual serotypes for serogroup 18 could not be determined due to the limitations of low-resolution PCR and cost constraints restricting use of reference Quellung and latex serotyping methods. Serotype 18C, which is a highly invasive serotype [18], and included in all three pneumococcal conjugate vaccines owing to its high prevalence in IPD globally [5], is the most likely responsible serotype in this serogroup, however. Although there are no definitive reports of cross-protection for serotypes 18A, 18B, and 18F, these serotypes are considered vaccine related by a number of investigators [19], [20]. The incidence of these serotypes has been too low to determine whether the 18C antigen in PCV provides cross protection. In both meningitis and non-meningitis IPD, individual NV serotypes did not cause a large proportion of disease. In other settings that introduced the 7-valent PCV, some increase in NV serotypes was seen a few years after vaccine introduction. In those settings, the serotypes noted to increase were generally those that were most common before PCV7 introduction [21], [22]. The most common serogroup result found among NV serotypes was 12F/A/44/46 (unresolved to individual serotypes). In countries where either PCV7 or PCV10 have been introduced, 12F was found to be a common NV serotype causing IPD [17]. Continued surveillance and improved PCR methods will be required to demonstrate if 12F will become a more common cause of IPD in the post-vaccine era.

Our results from adult IPD cases demonstrate that the greater proportion of serotypes in this population is covered by conjugate vaccines as well as polysaccharide vaccines (PPV23). However, due to paucity of adult samples in our dataset, this finding needs to be verified in larger samples from adults with pneumococcal disease. In other settings, use of PCV among young infants has reduced transmission of vaccine type pneumococci from children to adults, preventing disease in older populations [23]. Whether these indirect benefits of PCV will occur in settings such as Pakistan still needs to be measured.

Our work has several limitations. We were able to serotype pneumococci from only a selected few samples submitted for examination and, therefore, results may not represent all IPD. Our data contain very few cases of pneumonia, which is the more prevalent form of IPD. Serotype proportions therefore may not represent the true proportion in the population for nonmeningitis IPD. Moreover, use of alternative serotyping methods such as Quellung typing or latex serotyping could have improved our results; however, use of these methods was not possible due to cost constraints and insufficient CSF specimen volumes.

This is the first comprehensive pneumococcal serotyping data from Pakistan since the study by Mastro et al. These data provide a baseline with which to compare IPD rates and serotypes following the introduction and roll-out of PCV10 in Pakistan. We therefore recommend continued surveillance of IPD followed by pneumococcal serotyping for improved serotype-specific IPD surveillance. The data hold promise that PCV will target a large proportion of disease in the region.

## Supporting Information

File SI **Figure S1 & S2.** Figure S1 shows pneumococcal meningitis serotypes by year in children 0–59 years, 2005–2013 in Hyderabad and Karachi, Pakistan. Different colors represent numbers of meningitis cases seen for that year due to individual serotypes. Figure S2 shows pneumococcal sepsis serotypes by year in children 0–59 years, 2005–2013 in Hyderabad and Karachi, Pakistan. Colors represent numbers of sepsis cases occurring each year due to individual serotypes.
(DOCX)
