# Supplementary material for: Pneumococcal Serotypes and Serogroups Causing Invasive Disease in Pakistan, 2005–2013
Source: PLoS One. 2014 Jun 3;9(6):e98796. doi: 10.1371/journal.pone.0098796 (PMC4043782; doi:10.1371/journal.pone.0098796)
Supplement: File SI — Figure S1 & S2. Figure S1 shows pneumococcal meningitis serotypes by year in children 0–59 years, 2005–2013 in Hyderabad and Karachi, Pakistan. Different colors represent numbers of meningitis cases seen for that year due to individual serotypes. Figure S2 shows pneumococcal sepsis serotypes by year in children 0–59 years, 2005–2013 in Hyderabad and Karachi, Pakistan. Colors represent numbers of sepsis cases occurring each year due to individual serotypes. (DOCX) [file pone.0098796.s001.docx]

Figure S1: Pneumococcal Meningitis serotypes by year in children 0-59 years, 2005-2013 in Hyderabad and Karachi, Pakistan. Colors represent numbers of meningitis cases seen for that year due to individual serotypes.

NT= Non-Typeable pneumococci

Figure S2: Sepsis serotypes by year in children 0-59 years, 2005-2013 in Hyderabad and Karachi, Pakistan. Colors represent numbers of sepsis cases each year due to serotypes.
